# Case-studies of displacement effects in Dutch hospital care

**DOI:** 10.1186/s12913-020-05086-9

**Published:** 2020-03-30

**Authors:** Joost Johan Godert Wammes, Geert Frederix, Paulien Govaert, Domino Determann, Silvia Evers, Aggie Paulus, Niek Stadhouders, Patrick Jeurissen, Wija Oortwijn, Eddy M. M. Adang

**Affiliations:** 1 Radboud university medical center, Scientific Center for Quality of Healthcare, P.O. Box 9101, 6500 HB Nijmegen, the Netherlands; 2 Julius Center for Health Sciences and Primary Care, University Medical Center Utrecht, Utrecht, Netherlands; 3 Ecorys, Rotterdam, Netherlands; 4 Department of Health Services Research, Care and Public Health Research Institute (CAPHRI), Faculty of Health, Medicine and Life Sciences, Maastricht University, Maastricht, Netherlands; 5 Radboud university medical center, Health Evidence, Nijmegen, Netherlands

**Keywords:** Displacement, Opportunity cost, Priority setting, Rationing, Basic benefit package, Innovation, Health technology assessment

## Abstract

**Background:**

Under a constrained health care budget, cost-increasing technologies may displace funds from existing health services. However, it is unknown what services are displaced and how such displacement takes place in practice. The aim of our study was to investigate how the Dutch hospital sector has dealt with the introduction of cost-increasing health technologies, and to present evidence of the relative importance of three main options to deal with cost-increases in health care: increased spending, increased efficiency, or displacement of other services.

**Methods:**

We conducted six case-studies and interviewed 84 professionals with various roles and responsibilities (practitioners, heads of clinical department, board of directors, insurers, and others) to investigate how they experienced decision making in response to the cost pressure of cost-increasing health technologies. Transcripts were analyzed thematically in Atlas.ti on the basis of an item list.

**Results:**

Direct displacement of high-value care due to the introduction of new technologies was not observed; respondents primarily pointed to increased spending and efficiency measures to accommodate the introduction of the cost-increasing technologies. Respondents found it difficult to identify the opportunity costs; partly due to limited transparency in the internal allocation of funds within a hospital. Furthermore, respondents experienced the entry of new technologies and cost-containment as two parallel processes that are generally not causally linked: cost containment was experienced as a permanent issue to level costs and revenues, independent from entry of new technologies. Furthermore, the way of financing was found important in displacement in the Netherlands, especially as there is a separate budget for expensive drugs. This budget pressure was found to be reallocated *horizontally* across departments, whereas the budget pressure of other services is primarily reallocated *vertically* within departments or divisions. Respondents noted that hospitals have reacted to budget pressures primarily through a narrowing in the portfolio of their services, and a range of (other) efficiency measures. The board of directors is central in these processes, insurers are involved only to a limited extent.

**Conclusions:**

Our findings indicate that new technologies were generally accommodated by greater efficiency and increased spending, and that hospitals sought savings or efficiency measures in response to cumulative cost pressures rather than in response to single cost-increasing technologies.

## Background

In the Netherlands a broad agreement between stakeholders in the healthcare sector has been agreed on, among other things, maximum permitted budgetary growth (1.3% in 2019, decreasing to 0% in 2022, excluding wage and price adjustment). Budget pressure is further increased by the continuous introduction of cost-increasing health technologies. Decision makers, both at local and national levels, therefore need to make choices about how to spend their resources. At the national level, the Health Care Institute (ZINL) of the Netherlands advises the Minister of Health (MOH) on the contents of the basic benefit package. In 2016, ZINL advised the MOH not to reimburse two expensive drugs - Pertuzumab (Perjeta®) and Pembrolizumab (Keytruda®) - unless their cost-effectiveness would be improved, and budget impact would be less through price negotiations. The main argument was that, in current economic circumstances, reimbursement of these drugs could require *displacement* of more cost-effective services, resulting in a net loss of health benefits at the population level [1]. Based on this advice, the Minister negotiated lower prices with the manufacturers and decided that the drugs would qualify for reimbursement until the end of 2019 [2]. These negotiated prices have not been revealed in the public domain.

In England, Wales and Scotland, research into displacement has mainly focused on estimating the cost per QALY threshold [3, 4]. Little is known however, about displacement in practice, how displacement takes place and what services are displaced to accommodate new cost-increasing technologies. We know of few studies on the introduction of cost-increasing technologies. One Welsh study investigated how NHS commissioners accommodated financial ‘shocks’ originating from Technology Appraisals – recommendations on the use of new and existing medicines and treatments within the NHS - issued by NICE. They found that the ‘displacement assumption’ (existing services are displaced to accommodate cost-increasing technologies) generally did not hold; and that financial shocks originating from Technology Appraisals were generally accommodated by greater efficiency and increased spending. In addition, commissioners sought for savings or efficiency measures in response to cumulative cost pressures from multiple sources rather than in response to single Technology Appraisals [5, 6].

In the current research, we defined displacement as a process, a total of decisions and resulting consequences made in anticipation of, simultaneous with, or after the introduction of a cost-increasing technology, with a net negative health effect due to the introduction of the new technology. This displacement process includes priority setting at higher organisational levels and bedside rationing at lower organisational levels. Insight in displacement also requires exploration of the causality of resource allocation ((how) does one decision leads to another and the ultimate effects for individual patients). We defined priority setting as resource allocation decisions between different services, patient groups, or elements of care; whereas bedside rationing was interpreted as the effects of such decisions on individual patients [7]. Priority setting in general has been researched for many years, although relatively little attention has been paid to the impact on individual patients.

The aim of our study was to investigate how Dutch hospitals have dealt with the introduction of cost-increasing health technologies, and to explore the relative importance of three main options to deal with increasing cost pressure: increased spending, increased efficiency, or displacement of other services. Specifically, we aimed to analyse how stakeholders across all organisational levels of hospital care experience displacement, and how they understand or perceive resource allocation or displacement decision making. Six case-studies of different cost-increasing hospital technologies were conducted to understand similarities and differences between cases, and to investigate the mechanisms of displacement and how these relate to financial and organisational structure.

### Institutional background

In the Netherlands, nearly universal coverage for curative care is achieved through mandatory purchase of statutory health insurance from private insurers. The Health Insurance Act legally requires health insurers to provide a comprehensive nationally set benefits package. Decisions regarding the package rest with the Minister of Health, who relies on advice from the National Health Care Institute and its Healthcare Insurance Board [8, 9]. Coverage of prescription drugs is described in positive lists. Remaining service coverage is specified through an open specification with a general (functional) description of benefits, and restrictions are expressed in negative lists [10]. The great majority of services enter the health system without formal assessment through this ‘open’ specification.

The Health Care Institute uses four criteria to determine whether to reimburse a new health service: necessity, effectiveness, cost-effectiveness, and feasibility for implementation. Adoption of a technology is based on an integral assessment along these criteria, although the criteria are not used as knock-out criteria. The criteria are continuously refined and improved, and especially the cost-effectiveness criterion is debated. In 2006, the RVZ (government advisory body) argued that treatments with a cost-effectiveness ratio higher than €80.000/QALY should not be included in the basic benefit package. The RVZ also stated that the acceptable costs per QALY vary according to burden of disease and other factors, such as rarity of the disease [11]. In reality, treatments are rarely excluded from the basic package based on ‘unacceptable’ cost-effectiveness. In addition, besides the appraisal criteria, several other factors have played a role in defining the actual constituents of the basic benefit package, including the desire to control costs and public opinion [12].

The Dutch healthcare system is largely based on the principles of managed competition with little central planning. Health care purchasing is considered the centerpiece of the system and is the main instrument for stimulating efficiency. Insurers are supposed to prudently purchase care for their enrollees. In real life, insurers and hospitals mainly negotiate on volumes and prices, while quality of care plays only a minor role in these negotiations. In addition, insurers may decide not to contract a provider (selective contracting) but are required to offer adequate care for their enrollees. The great majority of hospital care in the Netherlands is reimbursed through payment products, similar to Diagnosis Related Groups (DRGs). More than 70% of DRG-prices are freely negotiable, the rest of the prices are regulated. A small part of hospital care is reimbursed through so-called add-ons. Add-ons are separate payments that have been developed for the reimbursement of expensive drugs and intensive care unit admissions.

From 2012 onwards, the Minister of Health has made sector agreements with providers and insurers that have effectively limited spending growth to 2.5% during 2012 and 2013, 1.5% in 2014 and 1% in 2015–2017. The agreement included an extra 1% spending growth allowance for primary care practices in 2014 and 1.5% in 2015–2017, provided they demonstrate that their services are a substitute for hospital care.

Insurers and hospitals negotiate prices and volumes on a yearly basis, guided by the terms of the sector agreements. Because of the sector agreements, hospitals and insurers de facto negotiate lump sum contracts with revenue ceilings as the most important provision. In addition to the ex-ante contracts with stipulated prices and volumes, a small part of hospital spending - for ‘non-steerable’ and very expensive services, including transplant care and expensive drugs - is carved out from the revenue ceiling and funded on a fee for service basis.

Hospitals and insurers negotiate 1) carved out contracts for expensive services, using add-on payments based on fee for service and without cap (in Dutch “nacalculatie”) and 2) ex ante a revenue ceiling contract based on prices and volumes (in Dutch “plafondafspraken”). Although the agreements allow for differentiation in percentages growth per hospital, the growth norm is used as a guiding principle for the negotiations. During the year, hospitals and insurers discuss new interventions and policy on a continuous basis. In autumn, new contracts for the upcoming year(s) are negotiated.

The carved-out contracts primarily include expensive drugs and expensive procedures. The budget for expensive drugs is not part of department budgets, but is a separate budget. Hospitals and insurers negotiate the volume and price of expensive cancer drugs, and sometimes also an ex ante determined capped budget. There are several requirements for carved out contracts, including guideline adherence, transparency, and no margins on the drugs.

## Methods

### Study design

We chose a multiple qualitative case study design to study displacement in the hospital sector of the Netherlands. Case studies are well suited to explore, deconstruct and reconstruct social phenomena, which we expected the displacement process to be. Our aim was to obtain the experiences and perspectives of a diverse range of stakeholders that have been involved in displacement decision making processes. We conducted six case studies to be able to understand similarities and differences between cases. Halfway through the project we organized an expert meeting with national experts (*N* = 9) in health economics and policy to discuss preliminary findings.

We purposefully chose six cost-increasing health technologies. First, a stakeholder meeting with our financial funder (ZINL) was held to identify case studies meeting a pre-specified set of criteria. In addition, we searched several (government) websites and explored cases through our personal networks. Apart from maximizing heterogeneity, interventions were required to meet the following criteria: 1) interventions should be generally considered cost-ineffective based on current Dutch standards as we were especially interested in decision making concerning new health technologies with disputable value to inform future decision making 2) interventions should be provided in hospitals 3) the reimbursement decisions should have been made at least 1 year ago, in order to be able to identify possible displacement effects 4) the intervention should have a relatively high budget impact. Based on these criteria, we chose intramural cancer drugs, robotic (Da Vinci) surgery, Left Ventricular Assist Device, endovascular aneurysm repair, population screening for colon cancer (which itself is strictly speaking not part of the hospital care, but the follow-up is), and expensive eye injections (Eylea and Lucentis). A short description of the interventions is given in Table 1.

**Table 1:** Description of the case studies

Health technology	Description
Left ventricular assist device (LVAD)	LVADs are devices for assisting cardiac circulation. They have been used from 1992 onwards as a ‘bridge to transplant’ for patients with advanced heart failure. During the years, the outcomes of the therapy have steadily improved, such that LVAD can be used as long-term therapy (‘destination’, LVAD is not followed up by a heart transplant).
Fenestrated endovascular aneurysm repair	In this procedure an expandable stent graft is placed within the aorta to treat aortic disease. This minimally-invasive technique is indicated for high-risk patients unfit for open surgery. Fenestrated and branched EVAR (FEVAR) are expensive due to its custom-made graft device.
Expensive oncolytics	In recent years, several relatively expensive oncolytics have been approved for inclusion in the basic benefit package, including pertuzumab, palbociclib, nivolumab, pembrolizumab, atezolizumab, and ibrutinib.
Eylea and Lucentis	Avastin, Eylea and Lucentis are all used for the treatment of various eye diseases. Eylea and Lucentis are both much more expensive than Avastin, but are equally effective for most indications. Eylea and Lucentis are indicated for patients for whom Avastin is not effective, and for patients with diabetic macula edema and vascular occlusion.
Population screening for colon cancer	In 2014, the Netherlands started population screening for colon cancer. People with positive test results are advised to get a colonoscopy in the hospital. Studies have shown that this surveillance is not cost-effective [13].
Robotic surgery	Robotic assisted minimally invasive surgery has been performed in the Netherlands since 2000, as an alternative to ‘pure’ laparoscopy or open procedures for various indications. Despite many studies, there is still no clear-cut evidence regarding the cost-effectiveness.

### Participant selection and recruitment

We purposefully selected key stakeholders to be interviewed for the case studies. Key stakeholders were initially identified from policy documents, websites, the media or from our network. These key informants all held senior positions in their hospital (department heads, professors, senior medical staff, department/division managers) and occupied key positions within their professional/scientific field (chairs of committees covering the case-study in the national scientific association). We then asked the initial key informants to suggest other participants (snowball sampling) with understanding of the (displacement) decision making surrounding the particular technology. We aimed to take into account geographic spread, to recruit a diverse set of relevant stakeholders, with different positions and responsibilities, per case study as well as per hospital within a case study. For example, we aimed to recruit informants from general, specialized as well as academic hospitals, and we recruited medical doctors, financial managers, sales managers, board members, and health care purchasers (insurers). In addition, we selected hospitals based on their level of scale up activity in a case-study; in other words, we recruited hospitals that were affected heavily financially by the entry of the case-study. All respondents held senior positions within their organisation. Participants were invited to participate in the study by e-mail. The invitation letter provided a summary of the aim and methodology of the study, as well as the time needed for the interview. We sent reminders when we did not receive a response within 2 weeks.

In total 84 interviews were conducted. Table 2 presents the characteristics of the respondents for each of the case studies. A minimum of nine interviews were held per case-study. Medical doctors were generally overrepresented among our respondents. However, in each of our case studies we interviewed a diverse range of stakeholders, with at least four distinct roles and responsibilities in the Dutch health system.

**Table 2:** Interview informants and roles per case study^a^

	Left ventricular assist device^b^	Fenestrated endovascular aneurysm repair^b^	Expensive cancer drugs	Eylea and Lucentis	Population screening for colon cancer	Robotic surgery
Medical doctors	5	8	12	8	8	6
Managers/directors	2	3	1	1	2	2
Professional scientific associations			1	1	1	1
Patient associations			1	1	1	1
Manager sales	1	3	1			1
Board of directors	1	1	5	4	4	2
Insurer		1	1	1	1	2
Hospital pharmacists			3			
Other			1		1	
Hospital respondents recruited from:	Recruited from three university medical centres	Recruited from two general hospitals, four university medical centres	Recruited from four general hospitals, five university medical centers	Recruited from two general hospitals, two specialized clinics, four university medical centers	Recruited from six general hospitals, four university medical centers	Recruited from six general hospitals, three university medical centers

^a^ Informants may be listed on several roles or columns. For example, a medical doctor may be a part-time member of the sales team of the hospital, or a member of the board of directors may have spoken about two or more case studies

^b^ We interviewed active members of the professional scientific associations (f.e. members of guideline committees), but not formally as representatives of the professional scientific association

### Data collection

The interviews took place between September 2016 and May 2017. All interviews per case study were conducted by one single interviewer. The interviews and analysis were undertaken concurrently and iteratively, in order to inform subsequent interviews. For example, we used subsequent interviews to verify and deepen statements in the former interview in order to better interpret statements, or to discuss aspects that the former respondents was not familiar with. The interview team met at least monthly during the duration of the study to discuss the findings and to coordinate ongoing work.

The primary aims of the interviews were to identify the main (financial) consequences of the introduction of the particular health technology for the department, hospital or insurer; and to discuss the choices and decisions that were made, as well as the reasons for making the decisions.

A semi-structured topic guide was used for the interviews, including the introduction process of the health technology; agreements and negotiation processes with third party payers; problems encountered (costs, time, facilities, etc.) due to the introduction of the health technology; what action was taken in response to the problems; the consequences for care provision and rationing; and views concerning displacement (the interview guide is presented in Additional file 1). Rationing was operationalized according to Klein’s rationing strategies, including rationing by denial, selection, delay, deterrence, deflection and dilution [7].

The topic guide was based on relevant literature and a pilot study and adapted based on the first five interviews. We made minor amendments to the topic guide for our interviews with hospital boards members and health insurers. During these interviews we discussed multiple case studies, and more time was devoted to displacement processes and priority setting in general.

The interviews were predominantly conducted face to face, at the respondent’s office, but twenty interviews were conducted by telephone. Interviews ranged 20–60 min and were audio-recorded and transcribed verbatim. Permission for audio recording was sought for and given in every case. The purpose of the interview and the general aim of the study were summarized at the start of each interview. We explained that neither findings nor quotes would be attributed to individuals or organizations.

### Data analysis and presentation

Transcripts were transcribed verbatim and analyzed thematically in Atlas.ti on the basis of an item list. This item list was derived from the semi-structured topic guide and literature. We made minor modifications to the topic list based on discussions within the broader study team. All analyses were performed by the interviewer who held the interview. In case of data ambiguity, we contacted the respondents to retrieve the meaning of a quote. For the purpose of inter-researcher reliability, the interviewers met regularly to discuss themes and data categories. In addition, at the start of the analysis, at least one transcript per case study was independently coded by two or more researchers and the results were compared. Any differences in data interpretation were discussed and resolved, and the approach was repeated until the researchers met satisfactorily levels of agreement.

**Fig. 1:**
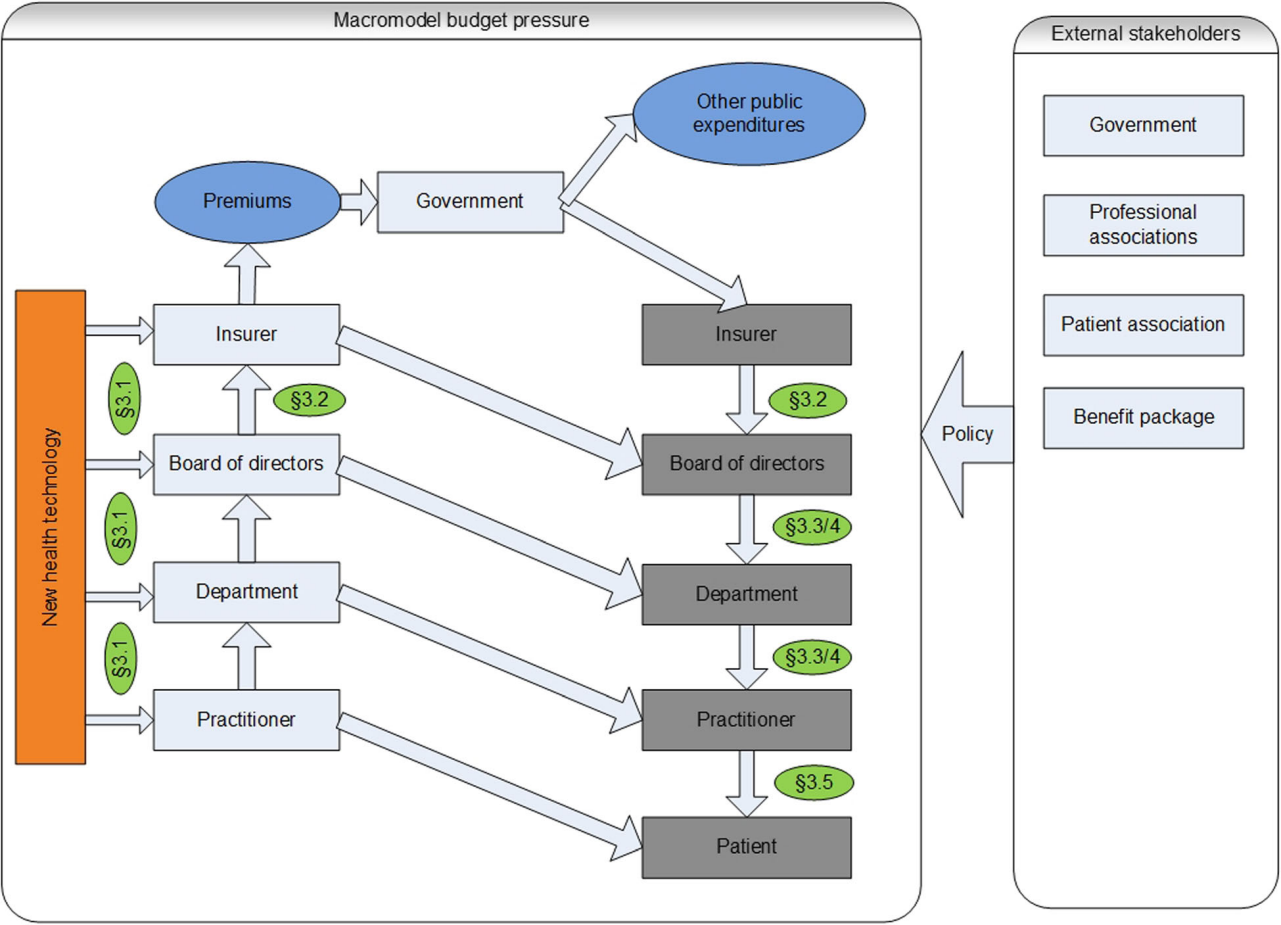
Macro model budget pressure, stakeholder model

We developed summary tables of the case studies according to the categories of the item list and compared the results across the case studies to identify systematic patterns of displacement. Based on this information, a narrative summary of the results was made. The results are presented according to the flow chart below (Fig. 1) which follows the budgetary flow in the health system and our interview guide. The arrows of the flowchart indicate how stakeholders can (re-)allocate the budget pressure, either upwards (left side), or downwards (right side). The green circles correspond with the paragraphs in the results section.

We first present respondent characteristics, and then discuss the introduction process of the health technology and actors involved; agreements and negotiations with insurers; problems encountered (costs, time, facilities, etc.) due to the health technology; decision making process in response to the problems; the consequences for care provision and rationing; and views concerning displacement.

## Results

Below the main findings of the interviews are presented. Paragraph 3.1 and 3.2 provide main contextual findings concerning the health technologies; which are essential for understanding the displacement mechanisms that will be outlined in paragraph 3.3 until 3.5.

### Entry into hospitals

An elaborate description of our research findings per case study is presented in Additional file 2. Entry into hospitals differed between cases. In most of the case studies, a wide range of stakeholders were involved in decision making processes, including health professionals, managers of hospital departments, board of directors, investment or drug committees, and in some cases also stakeholders from outside the hospital (medical societies, healthcare inspectorate, ZINL, other governmental agencies). However, in case of FEVAR, specialists and departments started experimenting with one or a few test procedures, before activities were scaled up after which stakeholders at a higher hierarchy level of the hospital were involved. LVAD, FEVAR, Lucentis and Eylea were all already used at considerable scale before they were formally included in the basic benefit package.

### Reimbursement, contracts and negotiation with insurers

According to the respondents, the reimbursement of cancer drugs is generally undisputed, and insurers will reimburse on basis of fee for service, as they fear for loss of reputation. However, indication extension during the year has led to budget overruns in some hospitals that were unable to negotiate extra money.

For remaining medical care, we found that negotiations about the capped revenue are parallel to, or subsequent to negotiations of the carved-out contracts. Hospitals generally prepare long lists of services they request additional funding for, and similarly insurers prepare lists of services they are willing to withdraw funding for. Based on the experience of our respondents, occasionally individual items on such lists are discussed and accepted or rejected. Hospital and insurer primarily negotiate a revenue cap, which is secondarily based on prices and volumes. Terms about specific services are not binding and may be exchanged for any other services. In addition, cross-subsidization (services are paid from the margins of other services) was widely reported.

### Problems encountered

The interviewees reported a wide range of problems they were faced with when the intervention was introduced. In case of LVAD and population screening for colon cancer, participants reported predominantly capacity problems (increased need for specialized personnel, operating room capacity, intensive care beds, colonoscopy capacity) and only limited financial problems. For the remaining four technologies, both capacity problems as well as financial problems were reported. Below the financial problems, and problems intrinsically related to displacement, are outlined.

#### Investment opportunities exceed the permitted growth

Both insurers and hospital management generally did not doubt the added value of most investment opportunities. However, it was clear that the associated total costs could not be accommodated in the current growth path. Many respondents argued that the increase in expensive drugs was at the expense of other services. It was hard to say however, at what expense exactly.

*“If that were not the case, then the rest of the negotiations might have been a lot easier. The expensive drugs are the elephant in the room”* – Board of directors.

Respondents generally pointed to the totality of budget increases, rather than to the growth of individual drugs or services. For example, FEVAR was *one of a range of* services contributing to the *cumulative* budget overrun. As a result, what could be observed is a competition between technologies and services for spending growth.

*“Instead, we do complicated things, like FEVAR-prostheses, complicated laparoscopic operations and so on. That costs twice as much, but our budget does not grow. So at the meso level of the department, there is a continuous fight with the Board of Directors.”* Vascular surgeon.

From the interviews it appeared that the degree of experienced cost pressure differed between settings, depending on the financial organisation of the hospital and negotiating power. Three respondents reported that the cost pressure in surgical departments/divisions was more severe than in cardiology/cardiothoracic divisions.

In addition, respondents argued that smaller hospitals face higher risks for cost pressure due to expensive drugs, as they were less likely to negotiate generous contracts with insurance companies. In the eye drugs case, specialized eye centres experienced heavier cost pressure, as they had less abilities for cross-subsidization or abilities to exchange services.

#### The distribution of flow of funds within a hospital is not transparent

Especially in larger and academic hospitals, many revenue sources exist, including innovations funds, education fees, research funds and others. According to our respondents, hospitals use internal funds through which the various revenue sources are reallocated (services were exchanged, or through cross-subsidization).

*“We work with a budget system. We negotiate on how that budget is built up, but it is up to the healthcare provider how to fill in that budget. A healthcare provider always has the possibility to reallocate the money somewhere else instead of to that DRG.”* Insurer.

Respondents reported a lack of transparency in the hospital’s internal financing. In the current system, negotiated DRG-prices may not represent real prices, and hospitals may lack insight in the costs of their DRGs. According to the respondents, negotiations rarely take place on intervention or technology level and are mostly based on hospital revenue deals. Consequently, the additional costs of an intervention or displacement effects are hardly visible.

*“The system is not so one-dimensional that such effects are immediately visible and you get a difficult conversation about the disposables [surgical instruments]. There are many possibilities and sources for substitution.”-* Surgeon.

Each hospital employed a sales team to negotiate contracts with insurers on behalf of the board of directors. Most of the managers and specialists we interviewed were not, or rarely/hardly involved in negotiations with insurers. In addition, managers and specialists noted that they were unaware of the specific provisions of the negotiated contracts. For example, three managers noted that they were unaware of negotiated prices for specific services. In contrast with that, most respondents noted that their department/hospital budget allowed certain maximum volumes for services. However, this maximum number of services was not necessarily a provision in contracts with insurers (external budget). Rather, this volume was used for internal planning in a decentralized internal budget, which was derived from the external budget. However, internal budgets may differ significantly from external budgets, as long as the revenue cap is not passed. In other words, provisions of the contract about specific services are not necessarily binding, and may be exchanged for other services, or allow cross-subsidization.

### Decision making processes, underlying reasons and contra-mechanisms to budgetary pressure

#### Decision making differs across types of financing, board of directors are central to decision making

According to our respondents, priority setting and rationing within hospitals differs depending on the type of service and type of budget. Expensive drugs requests are assessed by drug committees, before the Board of Directors are involved, who may negotiate additional budget from insurers. Because there is a separate budget for expensive drugs, the budget pressure was experienced at the higher managerial levels, not only by the department that uses the drugs. This budget pressure is (partly) accommodated by insurers and may be indirectly accommodated by departments in the hospital through lower department budgets.

In addition to the budget for expensive drugs, the hospital budget is cut into budgets for divisions and (sub-)departments. According to our respondents, departments and divisions are relatively free in how to spend this budget, but they are kept relatively strictly to this budget. They discuss their policy, budgets and activities with the board of directors on a regular basis.

*“If the cardiologists want to grow in the field of interventions, then maybe they should not grow in the area of the fast-track outpatient clinics. ”-* Board of directors.

Departments and divisions may submit business-cases to request additional funding. The board of directors (and sales team) are central to this decision making. They may decide to include the business-case in negotiations with insurers (*external* business-cases). Respondents noted that in exceptional cases (long waiting lists) hospitals have successfully negotiated extra funding. *Internal* business-cases are not discussed with insurers and may be rejected or funded from other sources. Most of the times, the board will request the departments to take austerity measures (see Contra-mechanisms). In each of the cases, the board of directors was involved in introductory decision making or growth.

#### Strategic considerations and key topics

A range of arguments were mentioned for introducing a technology, or to further invest in the growth of a particular service. In all case studies, patients were expected to benefit from the treatment. In addition, respondents argued that the technology was considered a key topic of the department and the hospital. Such emphasis on key topics can take many forms, and key topics were chosen at every managerial level (e.g. from high to low: cardiovascular centres, vascular surgery, aorta pathology). Generally speaking, such key topics receive more funding, at the expense of others.

*“In the coming years in particular the cardiothoracic and vascular domain will grow, maybe at the expense of others. That we say in other respects, that is no longer for us.”-* Board of directors.

Besides, several respondents pointed to competition between providers: providers were afraid to lose patients, or were afraid to stay behind technologically.

*“If we limit that flow of patients, then we will lose it, then they’ll look for someone else. Until today, that was one of the reasons why we accept the budget overrun.”-* Surgeon.

Some respondents doubted the benefits of the treatment and noted that a clear evidence base was lacking. Cost-effectiveness was rarely considered in introductory decision making. Besides, respondents argued the industry had ‘pushed’ the technology too much.

#### Contra-mechanisms

We asked the respondents how they dealt with the budget pressure of the new technologies. Respondents primarily pointed to their choices in the portfolio of their services. Insurers and the board of directors may request departments to stop providing services that can be provided elsewhere at lower costs.

*“Someone with a minor heart attack, and when treatment has gone straightforward, should simply be followed-up elsewhere.”* Cardiologist.

Some respondents doubted the budget impact of such measures, although it effectively reduced work load. Hospitals increasingly collaborate in this re-arrangement of service delivery, but the degree of collaboration differs considerably across hospitals.

A variety of other measures were mentioned to relieve budget pressure or capacity problems. This included efforts to reduce the price of LVADs, FEVAR-stents, and expensive drugs. Many doctors stated that they adhered to guideline recommendations more strictly than before, or that eligibility criteria for procedures or drugs were redefined. Besides, efforts were taken to reduce the length of stay or to technically improve services. Task rearrangements, substitution, e-health, and cuts in staff and beds were also mentioned.

### Displacement, and impact on regular care

We asked interviewees directly what measures were taken to accommodate the introduction of the technology, and which effects this had for regular care and for individual patients. In case of LVAD, respondents pointed to generous financing, and that problems primarily occurred due to capacity constraints. FEVAR was one of the services contributing to *cumulative* cost pressures, and in some hospitals FEVAR was rationed due to cost pressures from other services. Population screening for colon cancer was also rationed, but this was primarily due to shortages in GE-specialists. The additional costs for Da Vinci surgery were largely unknown, and cross-subsidized from other services.

The budget pressure of expensive drugs was accommodated by insurers and the board of directors, who redistributed this to the rest of the departments (horizontal reallocation). In the eye drugs case, rationing was widely reported, but cost pressure was only one of the several factors that necessitated rationing.

With few exceptions, there was consensus in our respondents that displacement, and efficiency/austerity measures were not causally linked to investments in technologies.

*“I cannot but remember that we had to cut costs and look for efficiency gains. But I cannot say that this really is at the expense or coincides with that Da Vinci. That is a permanent system to level the costs and the revenues.” -* Manager urology.

Although not necessarily related to the technologies, many respondents pointed to current pressures in Dutch hospitals, and the necessity to ration care. Below the most important mechanisms are outlined.

#### Rationing is usually the result of production caps and capacity problems

Many respondents noted that rationing was the result of cumulative pressure from several sources, including aging, reform in long term care, and technological innovation. Shortages in personnel and beds further complicated the situation. Occasionally, but not structurally, such capacity problems were related to austerity measures. Furthermore, respondents noted that individual services were rarely rationed, but that rationing occurred rather in larger organizational units, such as surgical divisions, or cardiovascular centres. Several respondents blamed the sector agreements and argued that insurers do not purchase enough care.

#### Hospitals primarily reduce accessibility in response to cost pressure

Respondents listed all rationing strategies; and rationing by delay was mentioned most frequently and was regarded the primary rationing strategy. In case of a budget overrun or capacity problems, the board of directors may request departments to reduce accessibility.

*“Yes, then we consult with the manager and the head of the department, and tell them to increase the waiting lists.” -* Board of directors.

Based on the experience of our respondent, rationing strategies were usually combined, especially rationing by delay and selection were often used in tandem. Furthermore, patients were prioritized on the basis of medical need: patients with cancer and acute patients got direct access, while non-acute patients were queued.

*“ If your operation room time is limited and you have to choose, the oncology patient is prioritized, and you are actually displacing the benign [non-cancer] patient.”*- Director surgical division.

Respondents noted that rationing strategies were used strategically to redirect patient flow. Hospitals focus their activities to more narrowly defined subpopulations or services. Consequently, patients with low complexity needs (*selection*) were *denied* access, or hospitals used long waiting lists (*delay*) for low complexity services.

Rationing by selection was often interpreted as a strategy to improve patient care, rather than a method to cut costs. For most respondents it was difficult to discern efficiency measures from rationing. Besides, respondents found it hard to identify the direct consequences for their patient’s health. Most respondents mentioned that competing hospitals had enough capacity to take over the patient flow. One potentially negative consequence for patients was increased travel time, and dissatisfaction due to their inability to go to the hospital of their first choice.

## Discussion

This paper presents how Dutch hospitals have dealt with the introduction of six cost-increasing health technologies. According to the experience of our respondents, the opportunity costs of cost-increasing health technologies could not unambiguously be identified; limited transparency in the allocation of funds downstream within a hospital contributed to this. Furthermore, respondents noted that the entry of new health technologies and cost-containment are two parallel processes that are generally not causally linked. In addition, the way of financing with a separate budget for expensive drugs may be pivotal in the Netherlands. According to the respondents, the budget pressure of expensive drugs is reallocated *horizontally* across departments, whereas the budget pressure of remaining services is primarily reallocated *vertically* within departments or divisions. Respondents’ hospitals have reacted to budget pressures primarily through a narrowing in the portfolio of their services, and a range of efficiency measures. The board of directors was found central in these processes, while insurers were involved only to a limited extent. Direct displacement of high-value care due to the introduction of new health technologies was not observed; respondents primarily pointed to increased spending and efficiency measures to accommodate the introduction of the technology. Rationing (primarily reducing accessibility) was observed mainly in response to cumulative cost pressures, production caps and capacity problems. In addition, respondents noted that patients were prioritized on the basis of medical need, cancer and acute patients were prioritized for non-acute patients, and that it was hard to identify the direct consequences for patients’ health.

Our analysis supports and builds on a relatively new field within health services research, a field that concerns identifying displacement effects as a response to the introduction of cost-increasing services, and estimating implicit threshold values to inform decision making concerning the basic benefit package. In line with Karlsberg Schaffer et al., our respondents noted that new technologies were generally accommodated by greater efficiency and increased spending, and that hospitals sought savings or efficiency measures in response to cumulative cost pressures rather than in response to single cost-increasing technologies [6].

One notable contribution of our research is that we, based on comparative analysis, identified two distinct pathways in which new technologies contribute to budget pressure. The first pathway includes funding for expensive drugs, which are explicitly appraised for inclusion in the basic benefit package. This budget pressure was said to be partly accommodated by insurers, and partly spread horizontally across several departments, albeit respondents found it hard to say to what extent departments accommodated the costs of (other departments) expensive drugs. The second pathway concerns funding for all other (non-pharmaceutical) technologies, which are rarely assessed by ZINL before entry, and the costs of which relate to the revenue cap. Our findings point out that the budget pressure of such technologies is generally reallocated vertically within the department or division. In addition, cross-subsidization and abilities to exchange funds were widely reported, and therefore insurers have limited abilities to control such spending. There is, however, generally a lack of clear-cut evidence about the value of the services. One risk in such implicit decision-making processes is that policy be based on arguments that may not be in line with maximizing population health. Indeed, personal factors (e.g. the “powerful” medical doctor) and competition between providers were named as arguments for approving a business-case.

### Implications for policy

Our findings indicate that new technologies were generally accommodated by greater efficiency and increased spending, and that hospitals sought savings or efficiency measures in response to cumulative cost pressures rather than in response to single cost-increasing technologies. In addition, rationing (primarily reducing accessibility) was observed mainly in response to cumulative cost pressures, production ceilings and capacity problems. Such problems are likely to worsen, given the newly established sector agreement with decreasing permitted budgetary growth (1.3% in 2019 to 0% in 2022, excluding wage and price adjustment). Possibly, as budgetary pressure increases, more drastic approaches may be applied to accommodate new technologies, which may well increase the opportunity costs of implementation, i.e., making health care as a whole less efficient. Furthermore, we showed that - albeit many stakeholders are involved - the introduction of non-pharmaceuticals is relatively uncontrolled, and that this may have undesirable effects. It is at this local level for non-pharmaceuticals where ad hoc decision making is ubiquitous, and sub-optimal use of scarce resources is very likely to occur.

Further policy may be aimed at strengthening or improvement of this local decision making, to carefully weigh benefits and costs of risky innovations with necessary stakeholders before scaling up of implementation. In the Dutch decentralized system, this may be most feasible, effective and is in line with the current sector agreements. Such policies will need all stakeholders to be involved, but especially board of directors of hospitals. In addition, some decisions may need to become more centralized. For example, legislators might consider whether the ‘open’ description of the benefit package for non-pharmaceuticals could become more ‘closed’, through adoption of cost-effectiveness as an additional reimbursement requirement in the open system. One option might also be to extend managed entry agreements to non-pharmaceuticals. Relevant stakeholders might join efforts to ‘guide’ the introduction of new technologies more prudently, for example through establishing minimum quality requirements, or to facilitate horizon scanning of new innovations. In addition, insurers might further develop their procurement policies to more effectively limit the entry of technologies with uncertain value, for example through concentrating the entry of innovations in selected hospitals via earmarked innovations funds for expensive or high risk innovations. Insurers could more strictly reimburse based on negotiated volumes for services, and limit possibilities for hospitals to exchange funds. In addition, hospitals could better align their internal budgets with their external budgets, and limit possibilities for cross-subsidization and abilities to exchange funds within their hospital. Finally worth mentioning is a look at other payment systems, like for example ‘pay for performance’.

### Strength and limitations

One major strength of our study is that we interviewed a wide range of stakeholders with diverse positions and responsibilities in the Dutch hospital sector, including macro, meso and micro perspectives on cost-increasing technologies. Especially this comprehensive approach adds to the present literature on this subject, that was mainly restricted to the macro or meso level. Although we claim a comprehensive approach it was not well balanced between stakeholders. For example, insurers were relatively underrepresented and we did not interview representatives from the (pharmaceutical) industry.

For most respondents it was difficult to discern efficiency measures from rationing by dilution, and it proved hard to identify the direct consequences of rationing strategies. As with all qualitative research, our findings may not necessarily extend to other settings. We purposefully identified six contrasting case studies, other case studies might have led to other results. Our analyses primarily concerned academic or relatively large hospitals, as most technologies enter the sector in these hospitals. Besides, our findings are dependent on the Dutch local context, most notably the way Dutch hospitals are financed and performances reimbursed. Social desirability or selective recall bias may have also influenced our findings. There may be no incentives for respondents to reveal displacement in interviews. However, our research methods were designed to cope with this problem, as we guaranteed that neither findings nor quotes would be attributed to individuals or organizations. Besides, we recruited a large number and diverse set of relevant stakeholders – also within hospitals - in order to verify and compare statements. However, we did not systematically identify similarities and differences between different groups of stakeholders.

More research is needed to identify displacement mechanisms in healthcare domains other than the hospital sector. Our research identified two distinct pathways of displacement effects, which are intrinsically linked to financing, and our approach may be fruitful in other countries as well. Furthermore, our findings once more point to the plethora of low-value service provision and lack of knowledge of the value of many services. More research is warranted in disinvestment of low-value services, and of (early) health technology assessment to prevent the introduction of promising, but nevertheless low-value services.

## Conclusions

Our findings indicate that new technologies were generally accommodated by greater efficiency and increased spending, and that hospitals sought savings or efficiency measures in response to cumulative cost pressures rather than in response to single cost-increasing technologies. We found that the opportunity costs of cost-increasing health technologies are not easily identifiable. Hospitals typically increase efficiency and disinvest in a range of low-value services, or services that may be provided elsewhere at lower costs, and decrease the volumes across the totality of their services. We found that the budget pressure of expensive drugs is reallocated horizontally across departments, whereas the budget pressure of remaining services is primarily reallocated vertically within departments or divisions. Decision making for entry of non-pharmaceuticals is often ad hoc and poorly informed by scientific evidence, which very likely results in sub-optimal use of resources.

## Supplementary information

**Additional file 1.** Interview scheme

**Additional file 2.** Results of the case-studies

## Data Availability

Not applicable.

## Abbreviations

ZINL: Health Care Institute
MOH: Minister of Health
NHS: National Health Service
NICE: National Institute Clinical Excellence
RVZ: Raad voor Volksgezondheid en Samenleving
QALY: Quality-Adjusted Life Year
DRG: Diagnosis Related Group
LVAD: Left Ventricular Assist Device
EVAR: Endovascular Aneurysm Repair
FEVAR: Fenestrated Endovascular Aneurysm Repair
