# Supplementary material for: Case-studies of displacement effects in Dutch hospital care
Source: BMC Health Serv Res. 2020 Mar 30;20:263. doi: 10.1186/s12913-020-05086-9 (PMC7106895; doi:10.1186/s12913-020-05086-9)
Supplement: Supplementary file 1 — Additional file 1. Interview scheme [file 12913_2020_5086_MOESM1_ESM.docx]

**Appendix 1. Interview scheme.**

Introduction

A consortium consisting of Radboudumc, Ecorys, Celsus, Maastricht University and Julius Center is conducting research into displacement effects in healthcare at the request of the National Health Care Institute. Displacement is described by the Healthcare Institute as follows: When assessing whether a treatment should be included in the insured package, it is assessed whether the health gain that can be achieved is in a reasonable proportion to the costs that have to be incurred (the cost-effectiveness). If this is not the case, the inclusion of the new treatment will be at the expense of the reimbursement of another treatment. This is based on the fact that the available money can only be used once. In order to map (potential) displacement at hospital level, we ask different care professionals and other stakeholders in the care for their opinion.

We would also like to discuss this with you during an interview of about 45-60 minutes. The goal is twofold:

- Find out what consequences the introduction of [case] has for your department and / or for the hospital and what choices were made as a result

- In addition, we are also curious about your vision of displacement in healthcare in general.

With your approval, the interview will be recorded using a voice recorder.

All data and information provided by you will be used confidentially and exclusively for the execution of this research. Do you have any questions or remarks so far?

Financing

First of all, we would like to gain insight into the way in which [case] is financed within your hospital.

• Could you please estimate the size of the patient group that is treated each year with [case] at your department / in the hospital?

• To what extent is [case] fully reimbursed by health insurers?

• Do you identify problems in the negotiation with health insurers about [case]?

Impact

• Has a horizon scan been carried out with regard to the introduction of [case] within your department / hospital?

• Has [case] led to identifiable problems at the ward / in the hospital?

• Has [case] led to certain choices that relate to regular care?

o Do you recognize one or more rationing strategies [a table with the six strategies was given] as a result of [case]?

o What do you think are the (possible) consequences of this rationing for patients?

o To what extent has [case] influenced the possibility of (previously planned) investments at your department / in the hospital?

• What are your expectations regarding the future deployment of [case]?

Involved actors

Based on literature research and initial interviews, we have developed a model, in which we map the way in which displacement may take place in healthcare. I would like to discuss this model with you.

• [Presenting figure 1]

o Who decides about the financing of [case] in your hospital?

o Where is the decision to introduce [case] within your organization most felt and by whom specifically?

o Do other hospitals in the region suffer from the choices that your hospital makes in relation to [case]?

o Do you notice shifts in care at national level that occur as a result of the admission of [case]?

• Which external parties are possibly involved in displacement in healthcare and how?

Displacement in healthcare

• In your opinion has [case] led to the displacement of other care?

• Has it ever happened that, due to the budget pressure, you were unable to provide the care that you would like to? If so, what specifically could you no longer do or did not want?

• Can you think of an intervention that entails a high risk of displacement?

Closing

• Do you have any other information or documents that are relevant to this topic?

• Do you have any suggestions regarding other colleagues that we could discuss?

• In due course, may we approach you again for any additional questions if we are further in the study?

• Do you have any questions or comments?

Thank you for your time and effort.
