# Supplementary material for: Case-studies of displacement effects in Dutch hospital care
Source: BMC Health Serv Res. 2020 Mar 30;20:263. doi: 10.1186/s12913-020-05086-9 (PMC7106895; doi:10.1186/s12913-020-05086-9)
Supplement: Supplementary file 2 — Additional file 2. Results of the case-studies [file 12913_2020_5086_MOESM2_ESM.docx]

Appendix 2: Results of the case-studies.

| Case study | Entry to the system | Reimbursement, contracts, negotiations | Difficulties or problems encountered | Decision making process and decisions, motivation, contra-mechanisms | Impact on regular care | Displacement? |
| --- | --- | --- | --- | --- | --- | --- |
| Left ventricular assist device | Due to improvements in the service, indications for ‘bridge’ and ‘destination’ were not mutually exclusive anymore.    Hospitals adopted destination therapy   1. Extending a end-stage heart failure program (starting in 2010) 2. Extending bridge to transplant program   Consequently, they could treat patients that could otherwise not be treated due to a shortage of donor hearts.  Departments or division wrote business-cases, or included growth of LVADs in their production plans for the coming year. Both were accepted by the board of directors.  In 2015 the indication ‘destination therapy’ was approved for the benefit package. | Board of directors used to grant departments a budget for LVADs from innovation funds, on the condition that the departments would publish in academic journals about the LVADs and would ensure LVAD would be adopted in the basic benefit package.  Later, LVADs were financed on the basis of fee for service, and carved out from the budget ceilings negotiations. Because LVADs are on list with ‘non steerable’ interventions, this led to no problems in negotiations.  Prices are set nationally, no volumes are negotiated, but estimations are made in advance for planning (attract additional personal etc, or that insurers can anticipate for the extra budget). | Increased need for:   - Specialized personnel. - Intensive care, cardiology beds. - Operation room capacity.   Other organisational and medical problems. | Departments wrote business-cases for board of directors for more budget and personnel.  National working group developed indication criteria and quality criteria.  Motivation:  LVADs are considered a priority topic by departments and hospitals.  Contra-mechanisms:   - Displace low complexity care from university hospital. - Efficiency measures, including reducing length of stay. - Negotiation to reduce the price of the LVAD. | Delay: other operative procedures are postponed.  Denial of patients with low complexity needs. | No *financial* displacement within the hospital, mainly because of the financing arrangement with relatively low risk for providers. In addition, the growth in number of LVADs stayed behind expectations. |
| Fenestrated endovascular aneurysm repair | Specialists and departments started experimenting early 2000s, activities were scaled up after initial success.  In 2013 FEVAR was approved for the benefit package.  Some hospitals started with FEVAR after the arrival of a new professor with experience in FEVAR. Besides, the technology improves so that patients can be treated that could other not be treated, and the technology is innovative/technically challenging, which is appreciated by the medical staff.  Competition between hospitals, hospitals are afraid to stay behind. | Some hospitals cross-subsidized FEVAR from other products.  Prices and volumes are negotiated between hospital and insurer, but these are not binding, may be exchanged for any other care, as long as the total expenditures do not pass the negotiated budget ceiling.  Thus, hospital and insurer primarily negotiate a total budget, which is secondarily substantiated by prices and volumes.  Some hospitals did not have any contracts with insurers about FEVAR.  Insurers rarely contract on basis of quality protocols, or review patient notes.  Departments/division wrote business-cases generally in reaction to budget overruns, rather than proactively. | Budget overrun for departments, divisions, cardiovascular centre. FEVAR was *one of the* services contributing to the *cumulative* budget overrun. FEVAR was a minor contributor, other services contributed more significantly to the budget overrun.  Increased need for facilities. | In some hospitals, extra budget was proactively negotiated within the department, division, hospital (overspend, from reserves) or from the insurer. In other hospitals, extra budget was negotiated (both with and without success) in response to the cumulative cost pressure.  In one hospital, operation room time was reduced to limit the number of surgical procedures and spending.  Motivation:   - Aorta pathology is considered a priority topic by departments and hospitals. - Competition between providers. - Less invasive is the future for surgery.   Contra-mechanisms:   - Negotiate lower prices for stents. - Displace low complexity care from university hospital. - Efficiency measures, including reducing length of stay. | Delay: waiting lists for surgery.  Denial or deflection of low complexity patients. | FEVAR was one of the services that contributed to cumulative cost pressures. In some hospitals, extra budget could be negotiated to accommodate this growth. In other hospitals, the growth of FEVAR was limited due to cost pressures from competing innovations.  Lower prices for stents reduce the likelihood of displacement.  Due to intransparent financing, no displacement effects could be traced. |
| Expensive oncolytics | Expensive oncolytics are reimbursed after EMA-registration, a positive evaluation of effectiveness by an established national oncologic commission (‘Commissie BOM’) and an assessment by the Health Care Institute.  Within hospitals, drug committees discuss new drugs and negotiate extra budget from the board of directors. | Expensive oncolytics are reimbursed using add-on payments, most often on the basis of fee for service, or ex ante determined capped budgets.  Hospitals negotiate with insurers about volumes and prices. In most hospitals, these negotiations are carved out from the negotiations guided by the budget ceilings.  There are several requirements for carved out contracts. Insurers require that hospital adhere to guidelines, be transparent about costs and outcomes, and allow no margins on the drugs (which used to be the case, and was spent on other care). | Concerns about future financial sustainability, exorbitant high prices, and high profit margins of industry.  Growth in spending for expensive oncolytics exceeds the 1% growth limit of sector agreements.  Thus, growth allocated to expensive drugs needs to be retained from the other services. | Negotiate additional budget from insurers, before and during the year.  Insurer and hospital first negotiate the total budget for the coming year (≈ budget-t_0_ +1%). Then this budget is cut to 1) all services that are carved out, and 2) other services, which are further cut in different segments of services.  Board of directors may request departments to cut costs. Ongoing cycle of austerity/efficiency measures within the hospital, in line with the policy of the organisation.  Motivation:   - Insurers are afraid for loss of reputation. - Oncology is considered a priority topic in hospitals, malign is prioritized above benign.   Contra-mechanisms:   - Negotiate lower prices, through for example collective purchasing. - Precision medicine. - Efficiency measures. - Displace low complexity care from university hospital. - Introduction of new drugs delayed to next calendar year. - Cuts in (support) staff. - Cuts in number beds. | Rationing of expensive oncolytics was not reported, however selection (more strict adherence to indication criteria than before) was.  Some treatments may be delayed, or patients were sent to other hospitals (deflection), however respondents were sure these had no negative impact on patient’s health.  Patients with an indication for expensive oncolytics were sent to and from other hospitals (deflection).  Investments were delayed. | Expensive oncolytics lower the growth of remaining services.  The budget pressure of the drugs was absorbed by board of directors and insurers, who redistributed this pressure to the rest of the departments.  Because of this indirect route and intransparent financing, it is difficult to causally link austerity measures or budget cuts, to the budget pressure of the drugs. |
| Eylea and Lucentis | Lucentis has been reimbursed from 2007 and onwards.  In 2012, ZINL advised to exclude Lucentis from coverage.  In 2012 and 2014 respectively Lucentis and Eylea were included in the positive list for add-on payments. The drugs were initially indicated for patients with age-related macula edema.  Eylea and Lucentis were also used for patients with diabetic macula edema and vascular occlusion. However, these indications were not included on the positive list for add-on payments until March 2015.  When new indications appeared on the positive lists during the year, the board of directors tried to negotiate extra budget for the drugs with the health insurers. | Eylea and Lucentis are reimbursed using add-on payments, most often on the basis of carved out contracts. In some hospitals however, these services counted for the budget ceiling.  In the past, eye injections were financed from DRGs (Avastin was mainly used for financial reasons) or from innovation funds.  Insurers and providers negotiate a percentage of Eylea/Lucentis of total eye injections, typically between 10-20%.  Insurers require transparency in the purchasing and use of the eye drugs. | Indication extension in the midst of the year led to budget overruns in hospitals where add-on payments for the drugs did count for the ceiled total budgets.  Especially specialized eye centres experienced cost pressure due to expensive eye drugs.  The number of patients and the percentage of patients requiring Eylea/Lucentis grows.  For insurers it is difficult to determine the right percentage of expensive injections.  Some insurers reimbursed more expensive eye injections than others.  Increase in number of patients, increased workload. | Renegotiation with insurer.  Internal redistribution of funds between departments by board of directors.  Cross-subsidisation from other services with high profit margin.  Board of directors control the volume and budget of departments.  Motivation:   - Board of directors decide about priority topics that are allocated a higher budget, compared to other departments/topics that are strictly kept to their budget.   Contra-mechanisms:   - Efficiency measures, including task rearrangements. - Displace low complexity care. - Improve adherence to indications, via committee for expensive drugs. | In exceptional situations, patients were selected on basis of insurance company. In addition, patients were selected on basis of medical urgency.  Deflection of patients that need expensive drugs because of inadequate budget.  Delay of non-acute care, or denial of referred patients.  Under spend on innovations/ investments/ maintenance. | Specialized eye centres experienced most cost pressure, presumably because they had less abilities for cross-subsidiation and internal redistribution of funds, or had less market power to negotiate carved out FFS contracts for expensive drugs.  Rationing by delay and selection were widely reported, however these were not causally linked to the eye drugs. |
| Population screening for colon cancer | Hospitals could choose to participate in the program. This was profitable due to 1) increased revenue for endoscopies 2) the extra patients because of resulting treatment of patients with positive test results. Hospitals are required to treat a minimum number of patients with colon cancer.  Adoption of population screening in the benefit package was based on an advisory report of the Dutch Health Council. | Screening endoscopies were initially reimbursed per service, not counting for the budget ceiling.  Later, hospitals and insurers explicitly negotiated about prices and volumes of screening endoscopies. | Undercapacity gastroenterology (GE) departments, shortage of GE-specialists.  Higher demand for colonoscopies than anticipated. | Internal redistribution of funds between departments by board of directors.  Departments and divisions are generally held responsible for their own budget and are given flexibility in how to spend the budget.  Departments, divisions and board of directors determine priority topics for which budgets are loosened.  Motivation:   - Priority topic of departments and hospital. - Colon cancer and oncology in general were considered key topics in some hospitals. - Colon screening was considered low complexity care in some academic centres. - Colon cancer patients were generally prioritized above patients with inflammatory bowel disease.   Contra-mechanisms:   - Hospitals communicate their capacity for screening endoscopies digitally to the national program. As such, they were able to reduce the volume of services and demand locally. - Displace low complexity care. - Regional partnerships. - Substitution. - E-health. | Deflection of patients to other regions.  Delay: waiting lists for regular endoscopies or GE-care in general.  Selection: in urgent cases patients were given priority and were exempted from the waiting list. Various alternatives for selection based on urgency.  Denial of request for second opinions. Academic hospitals deny low complexity patients. | Most of the reported problems or rationing were related to the pre-existing shortage of GE-specialists.  The screening program put further pressure on the waiting lists, but the waiting lists were generally not linked to adverse health outcomes. |
| Robotic surgery | The first Da Vinci platform was bought in 2000.  ZINL did assess Da Vinci, but not formally in- or excluded the service from the benefit package, because decisions about service coverage concern treatments for a given patient population, not the way the treatment is delivered.  Within hospitals, departments (primarily urology) requested the board of directors to buy the platform. | The procurement of the platform is financed from investment- or innovation budgets.  Insurers are not involved in and are not consulted for the purchase of the platform.  Hospitals and insurers mainly negotiate budgets, and make no specific agreements about specific procedures.  However, insurers may request a minimum number of procedures per year to guarantee high quality of care. | Lack of evidence for benefits of the platform.  DRGs for the laparoscopic procedure are not sufficient to cover the costs. Higher reimbursement has been requested, but were rejected by the insurers because of the lack of evidence concerning the procedure.  High price of the platform, because of the monopoly of the manufacturer.  Intransparency of costs of procedures in the platform, compared to conventional procedures. In general: little insight in the costs of the organisation.  Lower use of the platform than anticipated.  Increased costs of disposables. | Medical specialists, urologists in particular, requested the board of directors to buy the platform, and wrote a business-case. Board of directors approved the request or not.  Hospital tried to negotiate extra budget from insurers.  The laparoscopic procedures are cross-subsidized from other funds or from services with profit margin in the hospital.  Budget cuts for operation rooms, intensive care (primarily in budget for personnel).  Reduction in procedures that were considered non priority topics of the hospital.  Motivation:   - Marketing/competition between providers, to attract patients, providers are afraid to lose prostate cancer patients. - It was argued patients prefer robotic surgery over classical surgery. - Minimally invasive surgery was considered the future of surgery. - Oncology was considered a priority topic of the hospital. - Innovation/research was considered a core activity of the hospital. - Insurers aim to concentrate Da Vinci in a small number of high volume hospitals. - Larger hospitals generally have more abilities for cross-subsidisation and for equalisation, and stronger negotiation power.   Contra-mechanisms:   - Indication extension to recoup the purchase costs of the platform, or to attract patients in the region. - More strict adherence to indications. - Reduction in length of stay. - Increase in productivity. - Efficiency measures. | Delay: waiting times for departments that are not considered key topics of the hospital. Malign was prioritized over benign.  Opportunity costs for other investments/ innovations. | Lower use of the platform than anticipated has led to indication extension with questionable benefit for patients.  Due to intransparency of costs the additional costs due to the platform are unknown.  As a response to the budget pressure of the platform, hospital generally:   - reported the opportunity cost of other investments or innovation; - prioritized some key topics above others; - reduced investments in personnel (operation room, intensive care). |
